# Characterization of the Key Aroma-Active Compounds in Yongchuan Douchi (Fermented Soybean) by Application of the Sensomics Approach

**DOI:** 10.3390/molecules26103048

**Published:** 2021-05-20

**Authors:** Shuqi Wang, Yuan Chang, Bing Liu, Haitao Chen, Baoguo Sun, Ning Zhang

**Affiliations:** Beijing Key Laboratory of Flavor Chemistry, Beijing Technology and Business University, Beijing 100048, China; wsq930312@163.com (S.W.); changyuan960327@163.com (Y.C.); liubing960911@126.com (B.L.); sunbg@btbu.edu.cn (B.S.); zh_ningts@btbu.edu.cn (N.Z.)

**Keywords:** douchi, aroma-active compounds, AEDA, OAV, GC–MS, GC–O

## Abstract

Yongchuan douchi is a traditional fermented soya bean product which is popular in Chinese dishes due to its unique flavor. In this study, the key aroma-active compounds of Yongchuan douchi were characterized by the combined gas chromatography–olfactometry (GC–O) and gas chromatography−mass spectrometry (GC–MS) with sensory evaluation. In total, 49 aroma compounds were sniffed and identified, and 20 of them with high flavor dilution factors (FD) and odor activity values (OAVs) greater than one were screened by applied aroma extract dilution analysis (AEDA) and quantitated analysis. Finally, aroma recombination and omission experiments were performed and 10 aroma-active compounds were thought to have contributed significantly including 2,3-butanedione (butter, cheese), dimethyl trisulfide (garlic-like), acetic acid (pungent sour), acetylpyrazine (popcorn-like), 3-methylvaleric acid (sweaty), 4-methylvaleric acid (sweaty), 2-mehoxyphenol (smoky), maltol (caramel), γ-nonanolactone (coconut-like), eugenol (woody) and phenylacetic acid (flora). In addition, sensory evaluation showed that the flavor profile of Yongchuan douchi mainly consisted of sauce-like, sour, nutty, smoky, caramel and fruity notes.

## 1. Introduction

Douchi is a kind of traditional fermented food product which is wildly applied as a seasoning in Chinese dishes [1]. Douchi has a history of more than 2000 years, and it is one of the four traditional fermented bean products together with soy sauce, furu (fermented bean curd) and doujiang (fermented bean paste) [2]. The main material of douchi is soya bean or black soya bean, and it is rich in nutrition with beneficial levels of fatty acids, esters and amino acids such as ethyl linoleate [1]. Douchi not only as a dish but is also used in cooking processes, and plays an important role in the daily life of people. In the Tang Dynasty, douchi migrated eastward to Japan and became natto after development and transformation. Douchi then spread to Indonesia and other Southeast Asian regions and developed into the local food tempeh. In recent years, in-depth research on the physiological function and flavor of natto and tempeh made remarkable achievements [3,4], however, the competitiveness of douchi in the international market is gradually weakened due to poor quality, high salt content and unstable flavor [5].

At present, the study on the physiological function of douchi is a hot topic. Research shows that douchi has the function of lowering blood glucose [6], antivasoconstriction [7] and antioxidant activity [8]. With the increasing attention and understanding of its health functions and nutritional composition, douchi has started to become popular again. Moreover, flavor includes the taste and odor substances coming from both nonvolatile and volatile compounds, which play an important role in food quality [9]. The unique aroma and flavor of douchi is another important reason for consumers to choose it [1]. The taste properties of douchi depend on its compounds, which include organic acids, fatty acids and amino acids. Zhang et al. [10] reported that glutamic acid was high in both the natural and artificial types of Yongchuan douchi, which contributes to its umami taste, and the amount of bitter amino acids including isoleucine, leucine and arginine were higher in artificial Yongchuan douchi. Wang et al. [11] isolated and identified 131 kinds of volatile compounds from three kinds of commercial tempeh by simultaneous distillation and extraction with gas chromatography−mass spectrometry (SDE–GC–MS), mainly esters, acids and alcohols. In addition, only a small part of the volatile compounds detected by the GC–MS contributed to food flavor, namely the aroma-active compounds. Gas chromatography–olfactometry detection (GC–O) is a method for detecting the active substances of food flavor with the human nose as a detector emerging in recent years [12], which has been widely used in the analysis of food aroma. Researchers have previously studied the aroma active substances of fermented soybean products (such as soy sauce [13,14], fermented bean red curd [15,16] and soybean paste [17,18]). Chen et al. [19] used solid-phase microextraction (SPME) and GC–MS/O and found that 10 compounds, including 2-methylbutyraldehyde, 2,6-dimethylpyazine, 1-octene-3-ol, phenylethanol and phenyl ethyl butyrate were the key aroma-active substances in Liuyang douchi. Jelen et al. [20] detected 21 aroma-active substances in tempeh, among which 2-acetyl-1-pyrroline, 2-ethyl-3,5-dimethylpyrazine, dimethyltrisulfide, 3-methithiopropanal and other substances showed relatively high Odor Active Values (OAVs). However, the deep research on flavor analysis and the mechanisms of volatile compound formation of douchi is rare.

Yongchuan douchi with unique flavor is the representative of Mucor type douchi, which has strong and lasting aroma, a dark color and a soft texture favored by many people [21]. At the present stage, the aroma research of Yongchuan douchi is still carried out by single extraction method and GC–MS [10], and the research on its aroma-active substances is rarely reported. Therefore, the characterization of the aroma-active compounds of Yongchuan douchi is necessary. In this study, modern separation and extraction methods with sensory-directed flavor analysis was applied, such as gas chromatography–olfactometry (GC–O), gas chromatography−mass spectrometry (GC–MS), aroma extract dilution analysis (AEDA), odor activity value (OAV) calculation and aroma recombination and omission [22]. This research will produce insights into learning about the key aroma compounds in Yongchuan douchi, and help to study and adjust the formation pathways during Yongchuan douchi processing.

## 2. Results and Discussion

### 2.1. Aroma-Active Compounds in Yongchuan Douchi

To eliminate the influence of solvent, solid-phase microextraction (SPME) was used before SAFE (solvent assisted flavor evaporation) distillation. The SAFE distillate displayed the typical aroma of Yongchuan douchi. The volatiles were extracted by both SPME and SAFE and characterized through GC–O and GC–MS. A total of 49 aroma-active compounds were detected (Table 1). The aroma compounds were identified and characterized by comparing their RIs, mass spectra and odor descriptions with NIST library and reference standards, which included 11 acids, 7 aldehydes, 7 ketones, 7 pyrazines, 6 heterocyclic compounds, 5 phenols, 3 alcohols, 2 sulfur-containing compounds and 1 ester. There were two compounds with RI values of 2135 (herbal note) and 2380 (burnt note) that were temporarily unidentified. The various sensory properties of different kinds of aroma components indicated the complex flavor of douchi and that contribute to the unique flavor to Yongchuan douchi.

To figure out the importance of these odorants in the overall aroma profile, odor-active compounds in the douchi distillate were further investigated by applied aroma extract dilution analysis (AEDA) using GC–O. The analysis revealed 22 odorants (Table 2) with high FD factors (between 8 and 2048) which were recognized as aroma-active compounds of Yongchuan douchi. Four acids were obtained for acetic acid (pungent sour, FD = 2048), 3-methylbutanoic acid (rancid, FD = 512), 4-methylvaleric acid (sweaty, FD = 16) and 3-methylvaleric acid (sweaty, FD = 8). These organic acids provided a sour, rancid aroma to the douchi’s flavor and have been detected in Yangjiang douchi; among them, 3-methylbutanoic acid was identified as an active-aroma compound reported in Yangjiang douchi [23]. The odorants with relatively high FD factors of 2,3-butanedione (FD = 512) and γ-nonanolactone (FD = 512) were also recognized as significant components of douchi flavor. The γ-nonanolactone produced a milky note in the douchi flavor, and 2,3-butanedione was described as a fatty butter aroma, both of which have been discovered as aroma-active compounds in several fermented soybean foods [17,20]. Two sulfur-containing compounds with high FD values were another important class of aroma-active compounds found in Yongchuan douchi. The 3-(methylthio)propionaldehyde (FD = 256) is generally considered to have the aroma of roasted potato and the function of enhancing soya sauce-like flavor, which is one of the most common key aroma substances found in fermented soybean food products [14,16]. Dimethyl trisulfide (FD = 32) provided a typical sulfureted and onion-like aroma, and it was detected in doubanjiang [24] (a kind of fermented soybean paste). In addition, three phenols were found the aroma-active compounds and contributed a smoky note to the douchi’s aroma. P-cresol (burnt, FD = 64) and 2-methoxyphenol (FD = 16) were perceived as the smoky, burnt aromas, and the eugenol (FD = 16) was perceived as the woody, spicy aroma. Heterocyclic compounds generally have low threshold values and are powerful aromatic compounds in most heat-treated foods. However, most of the heterocycle compounds detected by the GC–O were not the aroma-active components in our sample. 2,3-Dihydro-3,5-dihydroxy-6-methyl-4(H)-pyran-4-one (FD = 256) gave the aroma of burnt sugar odor and was also discovered in Yangjiang douchi. Acetylpyrazine (FD = 16), which provided a roasted, popcorn-like note, was the only aroma-active pyrazine compound in Yongchuan douchi, and has never previously been detected in douchi or other soybean products. Maltol (FD = 16) is a common odorant in fermented soybean foods giving a pleasant, malty and caramel smell [13,14,17].

The odorants phenylacetic acid (FD = 1024), phenethyl alcohol (FD = 128), benzeneacetaldehyde (FD = 16), citronellol (FD = 16) and β-damascenone (FD = 16) showed floral, honey-like aromas, and all of them were detected in doubanjiang. In addition, 3-methyl-1,2-cyclopentanedione (FD = 16) and γ-nonanolactone (FD = 16) were characterized as aroma-active compounds contributing caramel and milky aromas, respectively, and the former was first detected in douchi product. The methyl palmitate (FD = 128) existed in doubanjiang, contributing the aroma of wax, and decanal (FD = 16) was detected in Yangjiang douchi, producing a green, soap-like note.

### 2.2. Quantitation of Aroma-Active Compounds and Calculation of OAVs

The concentrations of 21 aroma-active compounds were determined using calibration curves (Table 2). The 2,3-Dihydro-3,5-dihydroxy-6-methyl-4(H)-pyran-4-one was not quantitated because of the lack of standard compounds. Acetic acid (535.58 μg/g) and maltol (153.23 μg/g) were two dominant odorants in Yongchuan douchi. Although the rest of the odorants were in relatively small amounts of the douchi, it was still possible for them to produce huge contributions to the whole aroma profile. 

Aroma compounds with OAVs ≥ 1 are thought to affect the overall aroma of a sample. In our douchi sample, the OAVs of 21 aroma-active compounds were obtained (Table 3). Among them, 3-methyl-1,2-cyclopentanedione had an OAV < 1, and the remaining 20 compounds were thought to be the most significant aroma-active compounds for the overall sensory profile of Yongchuan douchi in this study. Dimethyl trisulfide and 2-methoxyphenol with OAVs of 8818 and 1317, respectively, might contribute greatly to the unique flavor of Yongchuan douchi.

### 2.3. Aroma Rcombination

**Figure 1:**
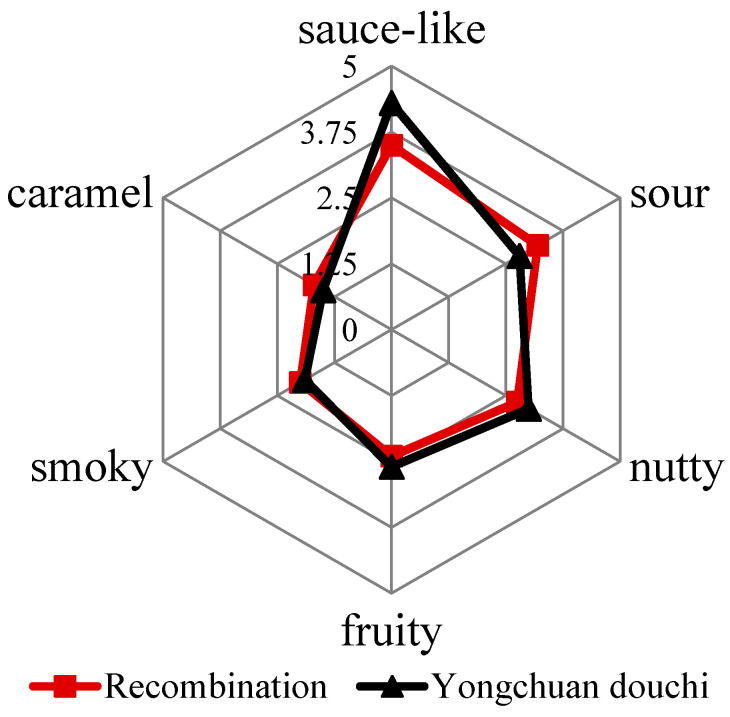
Aroma profile of Yongchuan douchi and recombinant model.

A comparison of the aroma profile of douchi and an aroma recombination was evaluated by sensory analysis to validate the screened 20 aroma-active compounds in Yongchuan douchi. The results were shown in Figure 1. The aroma of recombinant model showed good agreement with that of the original Yongchuan douchi sample, therefore, the sensory attributes (sauce-like, sour, nutty, smoky, caramel and fruity) that were produced by 20 key aroma-active compounds represented the overall aroma profile of Yongchuan douchi. However, the score gap of sauce-like note between recombinant sample and origin douchi sample was little big than others, and it might due to the lack of the 2,3-dihydro-3,5-dihydroxy-6-methyl-4(H)-pyran-4-one, which provided a burnt, caramel aroma and present in soy sauce.

### 2.4. Omission Experiment

To develop a deeper understanding on the aroma contribution of each key aroma-active compound, an omission analysis was performed using 20 models in which only one odorant was omitted. The triangle test results of every single compound are displayed in Table 3. The models omitted 2,3-butanedione, dimethyl trisulfide, acetic acid, acetylpyrazine and 2-methoxyphenol were identified by all or most of assessors, which indicated the significance of these key aroma-active compounds in contributing to the complete aroma of Yongchuan douchi. In addition, 3-methylvaleric acid, 4-methylvaleric acid, malton, phenylacetic acid, γ-nonanolactone and eugenol were also confirmed as key aroma-active compounds in our douchi sample by omission experiments.

### 2.5. Sources of Key Aroma-Active Compounds in Yongchuan Douchi

Fatty acid is one important class of key aroma-active compounds. Four fatty acids including acetic acid, 3-methylbutanoic acid, 3-methylvaleric acid and 4-methylvaleric acid were characterized as key aroma-active compounds contributing sour, rancid aromas to Yongchuan douchi. Although these acids had relatively high concentrations (acetic acid, 535.58 μg/g; 3-methylbutanoic acid, 14.21 μg/g; 3-methylvaleric acid, 2.04 μg/g; 4-methylvaleric acid, 3.55 μg/g) and OAVs > 1 causing rancid flavor in douchi, the flavor was pleasant and mild without an overpowering pungent aroma because of the delicate interactions of some volatiles with fruity notes [25]. As for the likely origins of fatty acids, the acetic acids are the metabolic product of the carbohydrate fermentation, 3-methylbutanoic acids are derived from leucine and valine degradation and acids with six or more carbons were from the lipolysis [25,26]. 2,3-Butanedione contributed butter, milky flavor, and had an OAV of 114, which was considered an important odorant in this study. Research showed the compound might originate from the lipolytic free fatty acids through enzymatic catabolism or antioxidation by the microflora in the sample [27]. 2-Methoxyphenol (OAV = 1317) with a savory, smoky aroma was thought to come from lignin-related phenolic carboxylic acid by thermal degradation [15]. Eugenol (OAV = 5) gave a woody, spicy odor, and the source was not clear.

3-(Methylthio)propionaldehyde (FD = 256, OAV = 229) and dimethyl trisulfide (FD = 32, OAV = 8818) were two sulfur-containing compounds found in the Yongchuan douchi sample. Sulfur-containing compounds were discovered in many fermented products and gave a great contribution to the whole aroma of samples, although they were found in relatively small amounts compared to others [28,29,30]. Dimethyl trisulfide may have derived from the methionine abundantly present in soybean by enzymatic or nonenzymatic breakdown [27]. In addition, heterocyclic compounds, including one pyrazine (acetylpyrazine, nutty note), one pyrone (maltol, caramel note) and one furanone (γ-nonanolactone, milky, coconut-like note), were characterized as predominant odorants in this study. The preparation of douchi includes several processes that can generate pyrazine compounds, which mainly involves cooking the soybeans and the drying and ageing processes. Alkyl pyrazines have been discovered generating naturally by the condensation of aminoketones as a result of the Maillard reaction and the Strecker degradation during the ageing process. Acetylpyrazine contributed a roasted, burnt aroma which has been identified in coffees [31,32] and wines [33], but if its origin is same as alkyl pyrazines remains to be explored. The maltol and γ-nonanolactone were two important substances for the flavor of many foods, and could be products of the Maillard reaction.

Benzeneacetaldehyde, phenylethyl alcohol and phenylacetic acid are regarded as the volatiles providing the aroma of flora and honey in flowers and foods. Benzeneacetaldehyde and 3-(Methylthio)propionaldehyde belong to Strecker aldehydes, most of which are generated by the decarboxylation and deamination reaction of corresponding amino acids during the fermentation of soybeans, and some of them are generated by the Millard reaction during the heating process [34]. Many studies have been carried out to elucidate the pathway for the synthesis of phenylethyl alcohol in bacteria and yeast. It was discovered that the yeast (Saccharomyces cerevisiae) can produce phenylethyl alcohol from phenylalanine via benzeneacetaldehyde and phenylpyruvate [35]. In addition to yeast, phenylethyl alcohol production can be through the benzeneacetaldehyde/phenethylamine route, as well as the pathway of transcinnamic acid and phenyl lactate. Both decanal (FD = 16, OAV = 2) and methyl palmitate (FD = 128, OAV = 4) have an aroma that is waxy and fatty, but the note of decanal is greener. Decanal in douchi might come from the changes in fatty acid, while methyl palmitate was formed by esterification. Citronellol (FD = 16, OAV = 33) might come from the precursor substances of isopentenyl pyrophosphate and dimethylallyl pyrophosphate via catalysis of geranyl diphosphate synthase [36]. β-Damascenone, (FD = 16, OAV = 470) was found in sparkling wines to contribute a sweet, honey flavor, and it is probably released from glycosidic precursors [37].

## 3. Materials and Methods

### 3.1. Materials

Yongchuan Douchi of original flavor was purchased from Waizumu Yongchuan Douchi Company (Chongqing Yongchuan Douchi Food Co., Ltd., Yongchuan, China). The time between Douchi production and experiments was about one month.

### 3.2. Chemicals

Ethyl ether and anhydrous sodium sulfate were purchased from Sinopharm Chemical Reagent Co., Ltd. (Beijing, China). Ethyl ether was freshly distilled before the experiments. Internal standard 2-methyl-3-heptanone (≥95%) and *n*-alkane (C6−C30, ≥99%) were purchased from Sigma-Aldrich (Shanghai, China).

Standard compounds used in analysis are as follows: 1-hydroxy-2-propanone (92%), 2,5-dimethylpyrazine (99%), dimethyl trisulfide (98%), acetic acid (99.7%), 2,3,5-trimethylpyrazine (98%), 2-ethyl-3,5(6)-dimethylpyrazine (99%), 3-(methylthio)propionaldehyde (95%), 2,3,5,6-tetramethylpyrazine (98%), isobutyric acid (99%), benzeneacetaldehyde (97.5%), isovaleric acid (99%), γ-caprolactone (98%), citronellol (95%), methyl cyclopentenolone (99%), maltol (99%), 2-acetylpyrrole (98%) and octanoic acid (98%); all were purchased from J&K Scientific Ltd. (Beijing, China). 2,3-butanedione (98%), 2-isopropyl-5-methylhex-2-enal (96%), 2-acetylpyrazine (99%), 3-methyl-1,2-cyclopentanedione (98%), phenol (99%), γ-nonalactone (98%), 5-methyl-2-phenyl-2-hexenal (95%), *p*-cresol (≥99.7%), eugenol (99%), methyl palmitate (99%), laevulinic acid (99%), benzoic acid (99.5%) and dihydro-4-hydroxy-2(3H)-furanone (96%) were purchased from Aladdin Biochemical Technology Co., Ltd. (Shanghai, China). 2,3-butanediol (>97%), 3-methylvaleric acid (>98%), 4-methylvaleric acid (>98%) and guaiacol (>98%) were purchased from TCI (Shanghai, China). Methyl heptenone (98%) and 2-phenylethanol (≥99%) were purchased from Macklin Biochemical Co., Ltd. (Shanghai, China). Decanal (97%), benzaldehyde (99%) and butyric acid (99%), were purchased from Adamas-beta Reagent Co., Ltd. (Shanghai, China). Damascenone (≥98%) was purchased from Sigma-aldrich (Shanghai, China). 5-methyl-2-furanmethanol (97%) was purchased from Fluorochem Ltd. (Derbyshire, UK). 2-pentadecanone (97%) was purchased from Alfa Aesar (Ward Hill, MA, USA), 2,6-dimethoxyphenol (99%) was purchased from Acros Organics (NJ, USA).

### 3.3. Aroma Profile Evaluation

Sensory evaluations were performed in a well-ventilated sensory laboratory with single testing booths under a constant temperature (25 ± 1 °C). Douchi samples (5 g of each) were put in Teflon vessels and displayed to a trained sensory panel of 10 subjects (2 males and 8 females, aged 22–48 years) recruited from Beijing Key Laboratory of Flavor Chemistry in Beijing Technology and Business University. All panelists had taken part in the training sessions for months to recognize the aroma profile of douchi. Six descriptors contributed the flavor of yongchuan douchi were selected by sensory evaluations and discussions among panelists: sauce-like, sour, nutty, smoky, caramel and fruity. The intensities of the aroma contributions of douchi were ranked from 0 to 3 (0, not perceivable, and 3, strongly perceivable) with steps of 0.5 units. Each sample was estimated three times by each panelist.

### 3.4. Isolation of the Volatiles: Solid-Phase Microextraction (SPME)

Crushed Douchi (3 g) was placed in a 10 mL SPME vial and 2 μL internal standard 2-methyl-3-hepanone (1.68 μg/μL) was injected immediately. The vial was put in an automatic constant temperature water bath (Yuhua Instrument Co., Ltd., Henan, China) and incubated at 50 °C for 40 min. The volatiles were then extracted at 50 °C continually for 40 min using a SPME sampler with the divinylbenzene/carboxen/polydimethylsiloxane (DVB/CAR/PDMS, 50/30 μm, 2 cm) fused silica coating fiber (Supelco, Inc., Bellefonte, PA, USA). After the extraction of volatiles, the coating fiber was immediately inserted into the GC injection port and desorbed for 5 min at 250 °C.

### 3.5. Isolation of the Volatiles: Solvent-Assisted Flavor Evaporation (SAFE)

Douchi (50 g) was crushed into paste and mixed with freshly distilled diethyl ether (200 mL), 2-methyl-3-hepanone (1.68 μg/μL, 100 μL) as the internal standard was added in mixture before extraction. Then, the volatiles were extracted by stirring at room temperature for 2 h and subsequently filtered. The residue was further extracted twice with diethyl ether (total 200 mL) after the filtration. The combined extracts were distilled by SAFE at 40 °C under the high-vacuum condition to isolate the volatile compounds from the matrix [38]. Then, the resulting distillate was dried over anhydrous sodium sulfate to remove water content and further concentrated to ∼5 mL using the Vigreux column (50 cm × 1 cm i.d.). The final concentrated fraction (approximately 500 μL) was obtained under a gentle flow of nitrogen steam and stored at −40 °C until next analysis.

### 3.6. Gas Chromatography-Olfactometry (GC–O) Analysis

GC–O analysis was by a gas chromatography-mass spectrometry (GC–MS) instrument (Trace 1310-ISQ, Thermo Fisher Scientific, Waltham, MA, USA) coupled with an olfactory detector (ODP3; Gerstel, Mülheim an der Ruhr, Germany). Two capillary columns with different polarities (TG-WAX and TG-5 capillary column) were used for separation volatiles. The temperature program had following settings: for the TG-WAX capillary column (30 m × 0.25 mm i.d., 0.25 μm film thickness; Thermo Fisher Scientific, Waltham, MA, USA), the initial temperature was 40 °C (kept for 3 min), then ramped to 120 °C at a rate of 5 °C/min (kept for 4 min), ramped to 200 °C at a rate of 5 °C/min, and finally ramped to 240 °C at a rate of 5 °C/min (kept for 8 min). For the TG-5 capillary column (30 m × 0.25 mm i.d., 0.25 μm film thickness; Thermo Fisher Scientific, Waltham, MA, USA), the initial temperature was 40 °C (kept for 1 min), then ramped to 100 °C at a rate of 3 °C/min (kept for 3 min), ramped to 280 °C at a rate of 5 °C/min (kept for 3 min), and finally ramped to 300 °C at a rate of 10 °C/min. Ultrahigh-purity helium was used as a carrier gas and delivered at a constant flow rate of 1.0 mL/min. Samples (1 μL) were injected into the injection port and the GC effluent was split by 1:1 through a Y-shaped effluent splitter at the end of the column to a sniffing port (250 °C) and a Mass detector. The mass detector conditions were as follows: ionization energy, 70 eV; the ion source temperature, 250 °C; and mass range, m/z 45–550.

### 3.7. Gas Chromatography–Mass Spectrometry (GC–MS) Analysis

A Thermo Fisher Trace 1300 gas chromatograph equipped with a Thermo Fisher ISQ single quadrupole mass spectrometer (Thermo Fisher Scientific, Waltham, MA, USA) was applied to acquire mass spectra of volatiles. TG-WAX and TG-5 capillary columns were applied to separate volatiles and the settings of program were same as the GC–O analysis.

### 3.8. Aroma Extract Dilution Analysis (AEDA)

Flavor dilution (FD) factors were determined by AEDA to measure the importance of odor-active compounds. The stored concentrated extract was diluted with diethyl ether in the following stepwise series: 1:2, 1:4, 1:8, 1:16…1:1024 according to the general AEDA method [39]. The series of dilutions were subjected to GC–O analysis on the TG-WAX column under the same conditions described above. The dilution was stopped until no odorants were perceived, and the FD factor of each odorant represented the maximum dilution at which the aroma-active compound could be detected by panelists. In order to eliminate potential gaps between panelists and ensure accuracy of the experiment, each dilution was analyzed by three experienced panelists (1 male and 2 females).

### 3.9. Identification and Quantitation Analysis

Aroma compounds detected by GC–MS and GC–O analysis were firstly identified by comparing their mass spectra with NIST 14 Library database (MS) and matching the odor descriptions (O) with standard compounds. The linear retention indices (RIs) for each volatile compound on the TB-WAX and TP-5 columns were calculated and compared to references values. A homologous series of C6−C30 *n*-alkanes were applied to determine the RIs of aroma compounds. The standard reference compounds (STD) were injected into the GC–MS to further verify the odorants.

Quantitative analysis for important aroma active compounds (with FD ≥ 8) was performed by diluting stock solutions to construct standard curves [28]. A certain amount of douchi sample (1 μL) was injected in GC–MS, and selective ion mode (SIM) was applied in quadrupole mass spectrometry. Five mixed standard solutions with different concentration ranges of 20 key aroma compounds were prepared, then each stock solution was serially diluted to six different concentrations and spiked with internal standard (1.68 μg/μL). The calibration equation of each odorant was obtained by plotting the response ration of standard compounds and 2-methyl-3-heptanone against their concentrations. Analysis was repeated in triplicate.

### 3.10. Odor Activity Value (OAV)

OAV was calculated by the ratio of the concentration and the respective threshold of each aroma compound. Due to the high water content of douchi, odor thresholds in water were used. The OAV values were considered to further evaluate the contributions of aroma-active compounds.

### 3.11. Aroma Recombination and Omission Experiments

Aroma recombination model was performed by mixing all quantitated key odorants (OAV ≥ 1) with their naturally concentrations in odorless matrix. The douchi odorless matrix was prepared by extracting the douchi sample (50 g) with diethyl ether several times until the residue was odorless. The model was compared to the original Yongchuan douchi by the sensory evaluation as described above.

Omission experiments for Yongchuan douchi were performed to further confirm the key aroma-active compounds. Twenty models were constructed by omitting only one odorant from recombination models. The sensory evaluations were performed between aroma-omitted models and complete reconstituted models through the triangle test [40]. The results were displayed as follows: very highly significant (distinguished correctly by more than 10 panelists, α ≤ 0.001), highly significant (distinguished correctly by more than 9 panelists, α ≤ 0.01) and significant (distinguished correctly by more than 8 panelists, α ≤ 0.05).

### 3.12. Statistical Analysis

The results were expressed as the mean ± standard deviation in triplicate analyses. One way analysis of variance was performed by SPSS 20.0 and significant differences were defined as *p* < 0.05. The radar chart was drawn by Origin 2018.

## 4. Conclusions

In this study, the key aroma-active compounds for Yongchuan douchi were successfully characterized. The GC–O identified 49 aroma compounds. Among them, 22 compounds (FD > 4) were selected by AEDA and 20 key aroma-active compounds (OAV > 1) were obtained by OAV calculation. The results were verified by an applied recombination analysis and omission experiments, which showed that 2,3-butanedione (butter, cheese), dimethyl trisulfide (garlic-like), acetic acid (pungent sour), acetylpyrazine (popcorn-like), 3-methylvaleric acid (sweaty), 4-methylvaleric acid (sweaty), 2-mehoxyphenol (smoky), maltol (caramel), γ-nonanolactone (milky), eugenol (woody) and phenylacetic acid (flora) contributed significantly to the aroma profile of Yongchuan douchi. In addition, these key aroma components resulted in the Yongchuan douchi with sauce-like, sour, nutty, smoky, caramel and fruity notes. Moreover, two unknown compounds presenting the flavor of herbal (FD = 4) and burnt (FD = 2) have not been characterized, and the 2,3-dihydro-3,5-dihydroxy-6-methyl-4(H)-pyran-4-one (FD = 256) still need to be obtained and evaluated in future work. Moreover, the results were only representative of Yongchuan douchi from Waizumu Douchi Company. If the key aroma compounds were the common content in most Yongchuan type douchi still needs to be confirmed.

The results in this study provide useful information for deep understanding of the volatile aroma compounds of Yongchuan douchi, which may be used as suggestions in technology development and quality control of douchi production.

## Figures and Tables

**Table 1:** Aroma compounds detected in Yongchuan douchi.

No.	Compounds	Odor ^a^	RI ^b^		Identification Methods ^c^	Extraction Method ^d^	
			TG-WAX	TG-5			
1	2-methyl butanal	malty	918		MS/RI/O/STD		SPME
2	2,3-butanedione	butter	969	<700	MS/RI/O/STD	SAFE	
3	1-hydroxy-2-propanone	herbal	1306		MS/RI/O/STD	SAFE	
4	2,5-dimethylpyrazine	nutty	1333	973	MS/RI/O/STD	SAFE	SPME
5	6-methyl-5-hepten-2-one	grassy	1344		MS/RI/O/STD	SAFE	SPME
6	2-Isopropyl-5-methyl-2-hexenal	herbal	1367	1167	MS/RI/O/STD	SAFE	SPME
7	dimethyl trisulfide	garlic	1386	1025	MS/RI/O/STD	SAFE	SPME
8	2-ethyl-6-methylpyrazine	roasted potato	1394	1060	MS/RI/O/STD	SAFE	
9	2,3,5-trimethylpyrazine	nutty skin	1416	1065	MS/RI/O/STD	SAFE	SPME
10	acetic acid	pungent sour	1419	<700	MS/RI/O/STD	SAFE	SPME
11	2-ethyl-3,5-dimethylpyrazine	nutty	1452	1141	MS/RI/O/STD	SAFE	SPME
12	3-(methylthio)propionaldehyde	roasted potato	1462	912	MS/RI/O/STD	SAFE	SPME
13	tetramethylpyrazine	chocolate	1476		MS/RI/O/STD	SAFE	
14	2-ethenyl-6-methylpyrazine	nutty	1495		MS/RI/O	SAFE	
15	decanal	green, soap-like	1502	1264	MS/RI/O/STD	SAFE	
16	formic acid	vinegar	1512		MS/RI/O/STD	SAFE	
17	benzaldehyde	almond	1527	1017	MS/RI/O/STD	SAFE	SPME
18	2,3-butanediol	fruity	1542	889	MS/RI/O/STD	SAFE	
19	3-methylbutanoic acid	rancid	1570		MS/RI/O/STD	SAFE	
20	acetylpyrazine	popcorn	1631		MS/RI/O/STD	SAFE	SPME
21	butyric acid	rancid	1639		MS/RI/O/STD	SAFE	
22	benzeneacetaldehyde	flora	1649	1100	MS/RI/O/STD	SAFE	SPME
23	3-methylbutanoic acid	rancid, fruity	1674	958	MS/RI/O/STD	SAFE	
24	γ-caprolactone	fruity	1702		MS/RI/O/STD	SAFE	SPME
25	5-methyl-2-furanmethanol	caramel	1723		MS/RI/O/STD	SAFE	SPME
26	citronellol	flora	1766	1288	MS/RI/O/STD	SAFE	SPME
27	3-methylvaleric acid	sweaty	1800	954	MS/RI/O/STD	SAFE	SPME
28	4-methylvaleric acid	sweaty	1808	1038	MS/RI/O/STD	SAFE	SPME
29	β-damascenone	flora, honey	1819		MS/RI/O/STD	SAFE	
30	methyl cyclopentenolone	herbal	1828		MS/RI/O/STD	SAFE	SPME
31	3-methyl-1,2-cyclopentanedione	caremelized	1839	1090	MS/RI/O/STD	SAFE	
32	2-methoxyphenol (guaiacol)	woody, smoky	1861	1151	MS/RI/O/STD	SAFE	SPME
33	phenethyl alcohol	rosy	1910		MS/RI/O/STD	SAFE	SPME
34	3-hydroxy-2-methyl-4H-pyran-4-one (maltol)	malty	1964	1181	MS/RI/O/STD	SAFE	SPME
35	2-acetylpyrrole	bread	1971	1124	MS/RI/O/STD	SAFE	SPME
36	phenol	plastic	2008		MS/RI/O/STD	SAFE	
37	2-pentadecanone	green	2019		MS/RI/O/STD	SAFE	
38	γ-nonanolactone	coconut-like	2031	1424	MS/RI/O/STD	SAFE	SPME
39	octanoic acid	rancid	2067		MS/RI/O/STD	SAFE	
40	5-methyl-2-phenyl-2-hexenal	nutty, cocoa	2076		MS/RI/O/STD	SAFE	
41	*p*-cresol	burnt	2085		MS/RI/O/STD	SAFE	
42	unknow1 ^e^	herbal	2135		O	SAFE	
43	Eugenol	woody, spicy	2168	1420	MS/RI/O/STD	SAFE	
44	methyl palmitate	wax	2215	1916	MS/RI/O/STD	SAFE	SPME
45	2,3-dihydro-3,5-dihydroxy-6-methyl-4(H)-pyran-4-one	burnt	2223	1212	MS/RI/O/	SAFE	
46	2,6-dimethoxyphenol	smoky	2265	1413	MS/RI/O/STD	SAFE	
47	levulinic acid	vinegar	2343	1138	MS/RI/O/STD	SAFE	
48	unknow2 ^e^	burnt	2380		O	SAFE	
49	benzoic acid	balsam	2458	1243	MS/RI/O/STD	SAFE	
50	phenylacetic acid	flora	2573	1320	MS/RI/O/STD	SAFE	
51	dihydro-4-hydroxy-2-(3H)-furanone	wax	2606		MS/RI/O/STD	SAFE	

^a^ The aroma sensed by human nose at the sniffing detector. ^b^ The Retention indices on different capillaries of TG-WAX and TG-5. ^c^ Identification methods of each odorants. MS: mass spectra; RI: retention indices; O: olfactometry; STD: standard compound. ^d^ Extraction methods of each odorants. SAFE: solvent-assisted flavor evaporation; SPME: solid-phase microextraction. ^e^ The compounds can be smelled by sniffing detector but cannot be identified.

**Table 2:** Concentrations, odor thresholds, FD factors and OAVs of 21 key aroma-active compounds in Yongchuan douchi.

No.	Compounds ^a^	Odor	Comcentration (mg/kg) ^b^	Odor Threshold in Water (mg/kg) ^c^	Linear Equations ^d^	FD ^e^	OAVs ^f^
1	2,3-butanedione	butter	0.685 ± 0.005	0.006	y = 0.099x − 0.0019	512	114
2	dimethyl trisulfide	garlic	0.088 ± 0.0002	0.00001	y = 0.186x − 0.0028	32	8818
3	acetic acid	rancid	535.577 ± 3.314	99	y = 0.0881x + 0.2166	2048	5
4	3-(methylthio)propionaldehyde	potato	0.103 ± 0.001	0.00045	y = 0.0551x − 0.0017	256	229
5	decanal	green	0.489 ± 0.001	0.245	y = 0.3267x − 0.0202	16	2
6	acetylpyrazine	popcorn	0.102 ± 0.0004	0.06	y = 0.3381x − 0.0101	16	2
7	benzeneacetaldehyde	flora	0.152 ± 0.001	0.0063	y = 0.5732x − 0.0017	16	24
8	3-methylbutanoic acid	rancid, fruit	14.213 ± 0.027	0.49	y = 0.2843x − 0.9345	512	29
9	citronellol	flora	2.070 ± 0.004	0.062	y = 0.5133x − 0.4318	16	33
10	3-methylvaleric acid	sweaty	2.040 ± 0.006	0.28	y = 0.057x − 0.0269	8	7
11	4-methylvaleric acid	sweaty	3.553 ± 0.006	0.81	y = 0.1827x − 0.2435	16	4
12	β-damascenone	flora, honey	0.006 ± 0.00001	0.000013	y = 0.5055x − 0.0013	16	470
13	3-methyl-1,2-cyclopentanedione	caramel	0.126 ± 0.002	0.3	y = 0.0179x + 0.0015	16	<1
14	2-methoxyphenol	woody, smoky	2.106 ± 0.003	0.0016	y = 0.7047x − 0.6626	16	1317
15	phenethyl alcohol	rosy	1.422 ± 0.002	0.5642	y = 1.0889x − 0.8019	128	3
16	maltol	malty	153.227 ± 1.2	9	y = 0.0062x − 0.0155	16	17
17	γ-nonanolactone	coconut-like	0.721 ± 0.002	0.027	y = 0.4415x − 0.1204	512	27
18	*p*-cresol	burnt	0.025 ± 0.00003	0.0039	y = 0.5119x − 0.0033	64	6
19	eugenol	woody, spicy	0.014 ± 0.0001	0.0025	y = 0.0955x + 0.0003	16	5
20	methyl palmitate	wax	8.920 ± 0.055	2	y = 0.0064x − 0.0025	128	4
21	2,3-dihydro-3,5-dihydroxy-6-methyl-4(H)-pyran-4-one	burnt	-	-	-	256	-
22	phenylacetic acid	flora	1.324 ± 0.004	0.464	y = 0.0344x − 0.0008	1024	3

^a^ The aroma-active compounds with high FD factors (FD > 4). ^b^ Concentration values of triplicates with standard deviations. ^c^ Odor threshold inquired from the book *Compilations of Odour Thresholds Values in Air*, *Water and Other Media*. ^d^ Linear equations were the fitted curve by peak area and corresponding concentration in Yongchuan douchi. ^e^ FD factors determined by AEDA. ^f^ OAVs were calculated by the concentrations and thresholds.

**Table 3:** Triangle test results of aroma-active compound (OAV > 1) by omission experiments.

No.	Compounds ^a^	N ^b^	Significant ^c^
1	2,3-butanedione	12	***
2	dimethyl trisulfide	11	***
3	acetic acid	12	***
4	3-(methylthio)propionaldehyde	7	-
5	decanal	7	-
6	acetylpyrazine	11	***
7	benzeneacetaldehyde	7	-
8	3-methylbutanoic acid	6	-
9	citronellol	5	-
10	3-methylvaleric acid	10	**
11	4-methylvaleric acid	10	**
12	β-damascenone	7	-
13	2-methoxyphenol	11	***
14	phenethyl alcohol	7	-
15	maltol	10	**
16	γ-nonanolactone	9	*
17	*p*-cresol	6	-
18	eugenol	9	*
19	methyl palmitate	4	-
20	phenylacetic acid	10	**

^a^ Key aroma-active compounds with OAV > 1. ^b^ Number of correct recognitions from 12 assessors. ^c^ Significance: *, significant (α ≤ 0.05); **, highly significant (α ≤ 0.01); ***, very highly significant (α ≤ 0.001); -, nonsignificant.

## Data Availability

The data presented in this study are available on request from the corresponding author.
